# Voices From the Frontline: A Qualitative Study of Nurses’ Experiences With Documentation and Its Influence on Patient Safety

**DOI:** 10.1155/jonm/6217086

**Published:** 2026-03-13

**Authors:** Reem Saeed Alghamdi, Monirah Albloushi, Marwa Alshareef, Hind Alshamry, Alaa Alabdulaal, Nadyah Alshakarah, Maryam Alrashidi

**Affiliations:** ^1^ College of Nursing, King Saud University, Riyadh, Saudi Arabia, ksu.edu.sa; ^2^ Operating Room, King Abdulaziz University Hospital, Jeddah, Saudi Arabia, kau.edu.sa; ^3^ College of Nursing, University of Hail, Hail, Saudi Arabia, uoh.edu.sa; ^4^ Coronary Care Unit, Prince Sultan Cardiac Center, Riyadh, Saudi Arabia, pscc.med.sa; ^5^ Family Medicine Clinic, King Saud University Medical City, Riyadh, Saudi Arabia, ksu.edu.sa; ^6^ Medical Unit, Prince Sultan Military Medical City, Riyadh, Saudi Arabia, psmmc.med.sa

**Keywords:** electronic health records, nursing documentation, nursing staff, patient safety, qualitative research

## Abstract

**Aim:**

This study aimed to explore nurses’ perceptions, experiences, and challenges related to clinical documentation and its influence on patient safety in Saudi Arabia.

**Background:**

Nursing documentation, a critical aspect of clinical practice, directly influences patient safety, care continuity, and legal accountability. Despite the increasing adoption of electronic health records, nurses continue to encounter numerous challenges in maintaining accurate and timely documentation, particularly in high‐pressure healthcare environments.

**Methods:**

A qualitative descriptive design was employed. Nine nurses with at least two years of clinical experience from various teaching hospitals in Saudi Arabia were recruited through purposive sampling. Data were collected through face‐to‐face semistructured interviews conducted between June and September 2023. Thematic analysis was conducted following an inductive coding approach supported by MAXQDA software to identify recurring themes and subthemes.

**Results:**

Three major themes emerged: (1) the perceived value of documentation as a legal and clinical tool; (2) barriers to effective documentation, including time constraints, lack of training, inconsistent practices, and weak handovers; and (3) nurses’ efforts to improve documentation quality despite feeling unheard in institutional decision‐making. The participants highlighted a shift in perception, viewing documentation not only as a protective measure but also as a key component of patient safety and care quality.

**Conclusion:**

The findings underscore the need for improved documentation training, standardized practices, and greater involvement of nurses in policy development. Addressing these gaps may enhance documentation accuracy, reduce burden, and promote safer patient outcomes.

## 1. Introduction

Nursing documentation, a fundamental component of patient care, is a written record of the care provided and serves as a tool for communicating accurate, up‐to‐date information among healthcare providers, enabling better decision‐making and care continuity [1, 2]. According to the World Health Organization (WHO) [3], effective documentation is crucial for preventing medical errors through ensuring ready accessibility of vital patient information to all healthcare team members. Accurate and timely documentation is essential for reflecting clinical assessments, interventions, and changes in a patient’s condition [4]. Many studies highlight that comprehensive documentation supports patient safety; it allows healthcare professionals to monitor clinical progress, evaluate treatment outcomes, and plan future care [2, 5, 6]. These records often include admission data, care plans, nursing actions, and patient responses [7, 8].

In addition to its clinical function, structured documentation improves communication clarity. For example, using standardized terminology, such as the International Classification for Nursing Practice (ICNP), helps avoid misinterpretation and ensures consistency [9]. Furthermore, adherence to structured documentation protocols has been shown to reduce adverse events linked to communication breakdown [10]. Conversely, inadequate documentation poses serious risks, including communication errors, delayed care, and compromised patient outcomes. Poor documentation has also been linked to longer hospital stays [11], increased patient mortality [12], and a higher incidence of medication errors [13, 14]. Additionally, legal liability, poor clinical decisions, and diminished care quality are some of the other consequences of poor documentation [15, 16].

Despite efforts to improve documentation standards, related challenges persist. The quality of documentation may be influenced by nurses’ experience, training, workload, motivation, and time constraints [17, 18]. Nurses spend between 17% and 40% of their workdays on documentation tasks, depending on the healthcare setting [19–21]. Although documentation is a critical aspect of nursing responsibility, it can be challenging, especially when compounded by burnout [22]. Pappa et al. [23] found that nurses experiencing high levels of burnout were more likely to make documentation errors or omit essential information during documentation.

To address these issues, electronic health records (EHRs) have been widely implemented to enhance documentation accuracy and accessibility [24]. EHRs offer numerous advantages, including cost reduction [25], improved clinical outcomes [26], and fewer medication errors [27]. They also provide structured templates that support standardized documentation and improve handoff communication during care transition [28]. Studies exploring nurses’ perceptions of EHRs have reported both the advantages and disadvantages associated with them [29, 30]. However, concerns include usability, training, cost, maintenance, and integration with the workflow, which may limit the full potential of EHR systems [31, 32].

The use of EHRs raises ethical and legal concerns among nurses. For instance, the “copy‐and‐paste” function, although designed to save time, can compromise patient safety [33], leading to diagnostic errors and inappropriate interventions [34]. In EHR‐related malpractice, 42% of issues were reportedly due to incorrect documentation and outdated patient information [35]. Research has shown that documentation errors can affect a nurse’s legal position and increase the risk of professional liability [36, 37]. Therefore, appropriate training, auditing mechanisms, and ethical guidelines are essential to ensure the safe and legal use of digital systems in nursing practice.

Several qualitative studies conducted in international settings have explored barriers to nursing documentation and their implications for patient safety. For example, Bjerkan et al. [38] identified individual, social, organizational, and technological barriers to safe documentation among healthcare professionals and students in primary care and older adult care settings. Their findings highlighted that documentation challenges compromise information exchange and continuity of care, thereby increasing risks to patient safety. Similar qualitative studies conducted in European and other Western contexts have emphasized documentation burden, system usability issues, and organizational constraints as key contributors to unsafe documentation practices [1, 22, 39]. However, documentation practices are highly context‐dependent and shaped by national regulations, accreditation requirements, workforce structures, and health information systems. As such, findings from these international studies may not be fully transferable to the Saudi Arabian healthcare context.

In Saudi Arabia, numerous studies have identified challenges that affect the quality of nursing documentation and compromise patient safety. Healthcare systems often face a shortage of nurses, resulting in heavier workloads and difficulties in maintaining accurate nursing care documentation, which then can compromise patient safety, legal accountability, and communication among healthcare professionals [40]. Moreover, lack of computer skills and inadequate EHR training hinders nurses’ ability to document effectively [41, 42].

Nurses’ attitudes and beliefs about documentation are also factors affecting documentation; one study assessing nurses’ knowledge, attitudes, and practices regarding documentation compliance found that 57.5% of the participants considered documentation to be a burden, 71.25% indicated that inconsistent documentation in patient files negatively affected patient outcomes, and 80% believed it was acceptable to delay documentation until after providing nursing care or performing procedures [43].

Although several studies have examined healthcare professionals’ perceptions of EHRs and found them beneficial, user‐friendly, and essential for improving patient care [41, 44–46], qualitative research in Saudi Arabia exploring how nurses perceive the importance of clinical documentation, the challenges they face, and the strategies they use to improve it has been limited. Furthermore, despite increasing investment in patient safety and digital health infrastructure in Saudi Arabia [47, 48], qualitative evidence elucidating nurses’ lived experiences with documentation and its influence on patient safety remains lacking. Therefore, this study was conducted to address these gaps by providing context‐specific insights into documentation practices within Saudi healthcare settings. Understanding these perspectives is essential to enhance training, standardization, and support in the clinical environment. This study aimed to examine nurses’ perceptions, experiences, and challenges related to clinical documentation.

## 2. Materials and Methods

### 2.1. Design

A qualitative descriptive design was employed to explore nurses’ perceptions and experiences regarding the role of documentation in patient safety. This approach was adopted to provide a clear description of the participants’ experiences and perceptions of documentation practices and their link to patient safety through the use of the participants’ own words. It is suitable when prior research is limited and the aim is to describe phenomena rather than generate theory or test hypotheses. Qualitative descriptive studies such as this summarize participants’ accounts with minimal interpretation, making the findings accessible and useful for practice, education, and policy [49].

### 2.2. Sample and Setting

Participants were recruited from large teaching hospitals with diverse clinical departments. Nurses with at least two years of experience in inpatient departments within a clinical setting were included, regardless of nationality, educational background, age, or gender. Nurses with fewer than two years of experience, those working in outpatient departments, and those holding nursing qualifications below a bachelor’s degree were excluded from the study. The settings included medical–surgical units, intensive care units, and outpatient clinics to ensure a comprehensive understanding of documentation practices across different contexts.

### 2.3. Data Collection Process

Data were collected from June to September 2023. Participants were recruited through WhatsApp and Twitter with an electronic invitation that detailed the study, the researcher’s contact information, and participants’ rights. This study used purposive sampling based primarily on hospital ward types to examine how ward‐specific clinical settings, workflows, and documentation systems, such as medical, surgical, and intensive care units, influence documentation practices and perceptions of patient safety. This emphasis on ward types enabled the capture of variation across clinical environments in which documentation practices differ. Interested individuals contacted the primary researcher for face‐to‐face semistructured interviews. All interviews were conducted by a member of the research team with prior training and experience in qualitative research methods, including semistructured interviewing. Nine nurses participated in the study, with recruitment continuing until data saturation was reached. Each interview, lasting between 45 and 60 min, was audio‐recorded with informed consent and conducted using a 12‐item semi‐structured interview guide (Table 1). The guide consisted of open‐ended questions designed to elicit comprehensive insights into nurses’ experiences with documentation, including educational preparation, routine documentation practices, perceived importance, and challenges encountered.

**TABLE 1:** Interview guide.

Question no.	Interview question
1	What motivated you to choose nursing as a profession?
2	Can you describe your typical daily routine at work?
3	What do nursing documentation systems mean to you in your professional practice?
4	Can you describe the training you received on nursing documentation during your university studies, internship period, and hospital orientation?
5	Can you describe your daily experience with documenting patient care?
6	How do you perceive the importance of nursing documentation in clinical practice?
7	How would you describe the relationship between the quality of nursing documentation and patient safety?
8	What methods do you use to document nursing care, and how satisfied are you with them? Have you attempted to improve or further develop your documentation practices?
9	What tools or systems do you use for nursing documentation?
10	What difficulties do you encounter during the process of documenting patient care?
11	What factors have contributed to the development of your nursing documentation skills?
12	Did you work during the COVID‐19 pandemic? If yes, how did the nursing documentation process differ during that period?

The initial items addressed participants’ backgrounds before focusing on their experiences, challenges, and the factors influencing their nursing documentation. The participants could respond in Arabic or English and were encouraged to express themselves freely. Each interview was conducted in a private setting to determine the participants’ choices. Before starting the study, the participants were briefed on the study, their rights, and the recording process. Field notes were taken during and after each interview to capture contextual details, interview dynamics, and researcher reflections to inform data analysis. Data collection continued until saturation was reached, ensuring that no new themes emerged.

### 2.4. Data Analysis

Interviews conducted in Arabic were transcribed verbatim in the original language. The transcripts were then translated into English by a bilingual researcher fluent in both languages and with expertise in qualitative research. To ensure semantic and conceptual equivalence, a second bilingual researcher independently reviewed the translated transcripts against the original Arabic versions. Any discrepancies were discussed and resolved through consensus to preserve the original meanings and cultural nuances of the participants’ responses. All interviews were analyzed concurrently with data collection, and no new themes emerged after the seventh interview. The final interviews confirmed existing findings. Data were analyzed by conducting an inductive thematic analysis following Braun and Clarke’s [50] six‐phase approach. The analysis involved familiarization with the data, inductive code generation, development of initial themes, review and refinement of themes, definition and naming of themes, and abstraction of final themes to represent participants’ experiences. Initial codes were generated inductively from the data without a predefined coding framework; these were compared and grouped into potential themes and subthemes related to nursing documentation and patient safety. Data collection and analysis occurred concurrently, and analysis continued until data saturation was achieved. MAXQDA software was used throughout the analysis to facilitate data management and organization.

### 2.5. Ethical Considerations

This study was approved by the King Saud University Institutional Research Board (KSU‐HE‐21‐301). The participants were fully informed about the research process and provided with contact information prior to participation. Participation was voluntary and anonymous, and participants had the right to decline to answer any questions or to request the removal of any information from the dataset. Pseudonyms were used to ensure participant confidentiality, and each interview was assigned a unique code. Personal information was not collected from participants. The transcribed files were securely password‐protected, and access was restricted to the research team.

### 2.6. Rigor and Data Trustworthiness

To maintain rigor in this study, several measures were implemented focusing on confirmability, dependability, credibility, and transferability. Confirmability was ensured by thoroughly evaluating the data, codes, and themes to ensure an accurate representation of participants’ experiences. The research team meticulously reviewed the transcribed interviews and repeatedly listened to audio recordings to verify the transcription accuracy. Reflexivity was maintained throughout the study, with researchers acknowledging and addressing potential biases to enhance objectivity. To establish dependability, the research lead served as an auditor, conducting detailed checks and reviews of all the interview transcripts. The identified codes and themes were cross‐validated to ensure their reliability. Additionally, an audit trail was maintained to document all methodological decisions and analytical steps, thereby ensuring transparency and consistency in the research process. Credibility was strengthened through a pilot interview conducted by the primary researcher to ensure clarity and comprehensibility of the questions. The research team reviewed the data and validated the emerging codes and themes. During the interviews, participants were given sufficient time to express their experiences and seek clarification to ensure a comprehensive understanding. Furthermore, member checking was conducted by sharing preliminary findings with the participants to validate their interpretations. Transferability was addressed through purposeful sampling, which ensured the inclusion of participants from diverse backgrounds to capture a broad range of experiences. Detailed descriptions of the research context and participant characteristics are provided to enhance transparency and facilitate the application of the findings to similar settings.

## 3. Results

A total of nine nurses were interviewed (Table 2). This qualitative study identified three main themes related to nursing documentation. The first theme focused on how nurses viewed documentation as a means of legal protection and a tool for monitoring patient care. The second theme focused on challenges such as time constraints, lack of training, frequent unit transfers, inconsistent practices, and weak handovers. The third theme highlighted nurses’ efforts to improve documentation despite feeling unheard while also recognizing the importance of supportive learning environments for developing documentation skills. Together, these themes underscore the need for improved training, consistency, and recognition of nurses’ input in documentation practices.

**TABLE 2:** The participants’ characteristics.

Characteristic	*n* (%)
Age (years)	
29 years and below	2 (XX)
30–39	7 (XX)
Gender	
Male	2 (XX)
Female	7 (XX)
Years of nursing experience	
2–5 years	3 (XX)
6–10 years	3 (XX)
More than 10 years	3 (XX)
Highest nursing qualification	
Bachelor’s degree	6 (XX)
Postgraduate degree	3 (XX)
Clinical unit	
Surgical wards	4 (XX)
Intensive care units (ICUs)	3 (XX)
Medical wards	1 (XX)
Others	2 (XX)
Type of documentation system	
Manual	3 (XX)
Electronic	3 (XX)
Both manual and electronic	3 (XX)

### 3.1. Main Theme 1: Perceived Value of Documentation

#### 3.1.1. Subtheme 1.1: Documentation as Evidence and Protection

Most nurses perceived documentation as providing evidence of accountability and patient safety. This finding indicates that documentation was seen as a means to ensure that care was provided and as a tool to protect both patients and nurses. Participants stated the following:

Using the ISBAR handover format helps prevent miscommunication by clearly documenting essential patient information, such as the patient’s name, file number, medical history, procedures, physicians’ orders, and recent laboratory results. Reviewing this form during handover helps the team understand the previous shift’s care and prevents errors in medication, procedures, or delays in treatment… (P1)

This is important because it protects me professionally and ensures patient safety. For example, with medications such as Syntocinon, I may forget whether the infusion rate was increased due to my workload, so accurate documentation is essential. (P5)

Some participants noted a shift in perception from initially viewing documentation as a means of protecting themselves to recognizing it as essential to ensuring patient safety and quality of care. One nurse stated the following:

We often hear that documentation is done to protect ourselves, not necessarily the patient. But once you begin working as a nurse, you realize that you’re not only protecting yourself—you’re also protecting your patient. (P3)

Participants noted that documentation protected them and demonstrated that the patient had received appropriate care. Some nurses considered that this evidence served to protect them legally and professionally, articulated as follows:

We do not mix information to avoid giving the wrong medication or intervention to any patient. We must document every intervention to prevent mistakes, as documentation is a legal record. Documentation proves that I am providing care correctly… (P6)

Documentation is now more readable and no longer handwritten, with less repetition. Previously, it was basic and lacked detailed health assessments, making it difficult to interpret, even in legal cases. Now, it is well organized, includes coded signatures, and clearly identifies who performed each task. (P4)

Therefore, most participants believed that undocumented care was equivalent to care not being provided.

The absence of documentation implies that the task or action was not completed… (P4)

When an intervention is not documented, it signifies that it was not carried out. (P7)

#### 3.1.2. Subtheme 1.2: Documentation as Ongoing Monitoring

Documentation was viewed as a legal record that supported continuity and quality of care. Most participants also viewed it as a tool to track and reflect on the patient’s condition and progress, as noted by one participant:

For example*, when a patient undergoes a procedure, we assess the patient beforehand and should reassess after the intervention for comparison. Sometimes this is not done, so the patient is recorded based only on the previous assessment, with no documented changes*. (P2).

Most participants also perceived documentation as recording interventions and patient progress in detail, tracking time, and capturing every minor detail of the patients’ experiences or events, as expressed by the following participants:

Every patient‐related event should be clearly documented, including what happened and the nursing interventions provided. (P7)

For instance, if I observe skin redness indicative of a pressure injury but do not document it, the condition may worsen. Early assessment and documentation of such indicators are essential because timely reporting can affect the patient’s health. (P9)

### 3.2. Main Theme 2: Barriers to Effective Documentation

#### 3.2.1. Subtheme 2.1: Time Constraints and Workload

Some participants felt overwhelmed by the many forms required by the Joint Commission International (JCI) and the Central Board for Accreditation of Healthcare Institutions (CBAHI), which delayed documentation. As one nurse noted, *“There are a lot of forms… the JCI and CBAHI require additional paperwork.”* (P1).

While another participant explained that nursing notes were first written on scratch paper and later formalized, time constraints made this process difficult. She shared, *“I jot things down first, then organize them into the nursing note, but the heavy workload often leaves me no time.”* (P2).

Similarly, a further participant found documentation to be time‐consuming because of the need to record everything, often with unnecessary repetition. She noted, *“Sometimes there’s repetition… it takes time, and that’s why some nurses write the physical assessment before actually doing it.”* (P3).

During the COVID‐19 pandemic, the participants noted increased documentation, especially in emergency settings. One participant shared, *“We used new visual triage forms with questions about animal exposure and respiratory symptoms like coughing.”* (P9).

Some participants expressed concern that documentation would take too much time, making it difficult to focus on patient care. This could often lead to unfinished tasks and added stress, as reflected in the following observations:

The value of documentation is often overlooked, leading to poor time allocation and added workload. (P4)

Documentation must be done within three hours; after that, it’s considered late. (P9)

Too many forms and paperwork make me feel patient safety is compromised. (P8)

In addition, some participants believed that staff shortages were a contributing factor that could lead to mistakes, as explained by the following participants:

Staff shortages and a heavy workload force me to keep documentation brief, which risks serious errors like double‐dosing of medication. (P6)

Caring for more than five patients due to staffing shortages affects documentation and leads to confusion. (P7)

Some participants identified several barriers to effective documentation, including limited time between cases, language challenges, lack of experience, and absence of supportive tools. Technical issues, such as distant computer locations and system shutdowns, were also noted as disruptions to workflow and efficiency.

Limited time, language, and lack of tools make documentation harder. (P8)

Using a computer far from patient rooms delays documentation. (P6)

Sometimes the system shuts down. (P9)

#### 3.2.2. Subtheme 2.2: Lack of Training and Preparation

Most participants reported insufficient documentation training during their formation as nurses, with education mainly focusing on care plans rather than practical documentation skills.

University training didn’t cover documentation well—most of the focus was on care plans. (P1)

We learned nursing diagnoses and interventions but not how to write nursing notes or why documentation matters. (P3)

Some participants highlighted gaps in documentation training, particularly for those completing bachelor’s degrees, compared with bridging students with prior clinical experience. Documentation exposure during internships also varied across hospitals depending on access to patient files and preceptor support, as expressed by the following participants:

As a bridging student, I was trained, but bachelor’s degree students lacked sufficient documentation training. (P7)

During my internship, access to patient files varied by hospital; learning depended on my initiative and preceptor support. (P1)

As trainees, we weren’t allowed to document—only to handle the data file. (P4)

Most participants described their three‐month orientation as heavily reliant on preceptor support, which varied in terms of quality and consistency. Many felt unprepared owing to their limited hands‐on experience during training, making the transition to clinical practice difficult, as stated by the following participants:

Orientation depends on the preceptor, but training wasn’t effective—we only observed, without practicing. (P5)

My preceptor followed my schedule, helped with care plans and handovers, and guided me through documentation. (P1)

Orientation covered documentation and corrections, but these skills weren’t taught during training—only learned on the job. (P6)

#### 3.2.3. Subtheme 2.3: Unit Transfers and Shift Handover

Most participants reported difficulties documenting when reassigned to unfamiliar units, such as pediatrics or postpartum care, owing to differences in procedures, responsibilities, and lack of experience. Variations in documentation styles and tools across hospital units further complicated the process, as stated by the following participants:

Working in pediatrics was challenging; I was used to adult care, and I lacked guidance, especially with medication calculations. (P7)

In postpartum care, we handle both mother and baby, but we’re not trained to document this type of care. (P8)

Each unit has a different documentation style: the emergency department uses brief notes, whereas medical units require longer entries. (P6)

Color‐coding and formatting vary across units, raising patient safety concerns due to the lack of standardized tools. (P3)

Accurate documentation is vital for safe handovers, but challenges during shift changes can affect the continuity of care and patient safety, as explained by one nurse:

I work 12 h shifts and arrive 30 minutes early for the handover. Before going to the bedside, we often complete unit tasks, such as checking the crash cart or intubation trolley and documenting the results. (P4)

Others noted that while tasks could be relatively uncomplicated, the volume of such tasks led to fatigue and missed breaks, often requiring them to stay late.

Too much paperwork—no time to eat or pray. We stay up to 1.5 h extra. (P5)

When my patient was critical, I couldn’t document until after the shift. (P8)

Some participants felt that the pressure to complete documentation led some nurses to prioritize paperwork over patient care, potentially compromising safety, as noted by one participant: *“Not all nurses have the same priorities—some focus on completing documentation before the end of the shift, even before attending to patient care.”* (P3).

#### 3.2.4. Subtheme 2.4: Lack of Standardized Documentation Practices

Many participants noted that unclear formats and inconsistencies in documentation tools made the process complex and error‐prone. Most nurses had reflected on the challenges of the documentation systems. Some relied on manual notes to manage time during busy shifts, whereas others emphasized documenting all patient details, even minor ones, to ensure accountability. Inconsistent formats and the dual use of paper and electronic systems added to the workload, as indicated by the following nurses:

I document anything risky and report it to protect myself. (P5)

I write on paper first to save time, then transfer to the system later. (P6)

We record everything—even small or routine details, including conversations. (P9)

Electronic documentation was limited to specific units such as critical care, while other units still used manual methods, as one participant noted: *“Most wards use manual documentation; critical care uses electronic.”* (P1).

Participants noted differences between electronic and paper‐based documentation. Electronic systems were considered efficient, organized, and reliable, as observed by one participant: “Electronic records are clearer, quicker, and legally stronger than old handwritten ones.” (P4).

However, another participant noted a preference for paper due to ease of use and limited computer access: *“Paper is easier, computers are slow and not always available.”* (P8).

### 3.3. Main Theme 3: Efforts to Enhance Documentation Quality

#### 3.3.1. Subtheme 3.1: Lacking a Voice and Being Dismissed

Most participants felt that their concerns regarding documentation, such as time burden, redundancy, and unclear structure, were often ignored. Junior and bedside nurses reported that they were not listened to and that suggestions rarely led to action, highlighting a lack of involvement in decision‐making processes, as noted by the following participants:

…they don’t listen to the junior staff. We always raise the issue of time‐consuming form‐filling whenever the quality team asks but nothing changes. (P1)

I joined a chart review committee and raised concerns about structure, color coding, and redundancy. No action was taken, but I felt I did my part as a bedside nurse. (P3)

There’s no chance… there’s no time even to make suggestions, and even if you have one, the process is extremely long, difficult, and often dismissed. In the end, your voice just isn’t heard. (P5)

#### 3.3.2. Subtheme 3.2: Supportive Learning and Development of Skills

Most participants highlighted the value of ongoing learning, mentorship, and a structured orientation in building strong documentation skills. Study days, preceptor feedback, and observing senior staff were considered key to improving accuracy and professionalism.

Attending study days and being observed by team leaders helped ensure proper completion of documentation. (P1)

If I have difficulties, I can ask the experienced person who wrote the documentation. (P2)

I was fortunate to have a supportive clinical instructor who reviewed my files and gave helpful feedback that improved my documentation. (P3)

When you read and see how others write, it helps you learn how to prioritize. (P8)

Interacting with senior staff helps improve documentation skills through observation and learning new techniques. (P9)

Structured orientation programs that included well‐prepared templates, such as “Perfect Nursing Notes,” helped set clear standards, which one nurse highlighted as follows:

During orientation, we were given well‐prepared templates like the “Perfect Nursing Notes” template, which helped us learn how to document professionally and in an organized way. (P5)

Time management, personal initiatives, and exposure to different documentation styles were considered key strategies for improving performance, as reflected in the following observations:

I try to manage my time, so the key factor that helps me improve my nursing documentation is time management. (P6)

…most learning came from personal effort—I might go on YouTube to find something useful. (P5)

There was also a call to enhance documentation training at the university level, as some participants felt that their foundational education was often insufficient. One participant stated, *“…documentation needs to be practiced and taught more intensively at the university level.”* (P7).

Another participant felt that quality improvement efforts focused too heavily on documentation, draining nurses’ energy and diverting attention from direct patient care: *“I hope quality efforts focus more on patient care than on paperwork, which drains nurses’ energy.”* (P1).

## 4. Discussion

This qualitative study explored nurses’ experiences with documentation tools and practices in Saudi Arabian healthcare settings, using in‐depth accounts from nine nurses across various institutions. The findings offer important insights into how documentation is perceived and the challenges it raises in everyday nursing work, particularly in relation to patient safety. The participating nurses described multiple challenges, including technical and system‐related concerns, which align with the WHO patient safety strategy emphasizing patient engagement, training, human factors, and the reduction of errors across diagnosis, medication, and care transitions [51]. These challenges are discussed in the following subsections.

### 4.1. Documentation as a Routine Requirement

The nurses viewed documentation as more than a routine administrative requirement. They described it as central to professional accountability, legal protection, and continuity of care, with accurate and timely documentation minimizing miscommunication, enhancing clinical decision‐making, and supporting patient safety [38]. Over time, perceptions shifted from viewing documentation primarily as a defensive practice aimed at legal safeguarding to a more patient‐centered understanding informed by clinical experience. This suggests that professional socialization and experiential learning are critical in shaping nurses’ conceptualization of documentation as a communicative and safety‐oriented tool that underpins interprofessional coordination. This interpretation aligns with broader literature indicating that high‐quality documentation supports multidisciplinary communication, reduces duplication of interventions, and promotes responsive, evidence‐based care plans [1, 52]. Documentation also contributes to institutional accountability and quality improvement by providing data that can inform policies, audits, and system‐wide learning [39], underscoring the importance of a strong documentation culture for transparency, coordination, and patient‐centered outcomes.

### 4.2. Training and Preparedness for EHRs

In this study, differences were observed between bachelor’s degree nurses and bridging students, which suggests that educational pathways may shape how nurses perceive and enact documentation. Bridging students, who typically enter with prior diploma‐level practice experience, may have greater exposure to paper‐based or locally developed documentation routines but less structured preparation in standardized or electronic documentation systems. In contrast, nurses with bachelor’s degrees may receive more formal theoretical input but have limited experience with real‐time, practice‐based documentation. These differing trajectories may help explain variations in confidence, perceived readiness for EHR use, and vulnerability to documentation‐related anxiety, reinforcing the argument that documentation competence is influenced by both curricular design and prior practice context [5, 53]. Participants also highlighted persistent gaps between academic preparation and the demands of clinical documentation, reporting limited exposure to practical documentation skills and real‐time charting, especially in EHR use, which contributed to feelings of unpreparedness and anxiety at entry to practice. Taken together, these findings suggest that documentation competence is embedded not only in technical proficiency but also in confidence and professional identity, particularly in transition‐to‐practice periods. This interpretation supports previous research that has identified a persistent underemphasis on documentation skills in nursing curricula and has linked inadequate training to documentation errors and inefficiencies [53]. In line with evidence showing that adequately trained nurses are 4.2 times more likely to document routine practices than inadequately trained nurses [5], these results highlight the need for structured, experiential training in documentation as a core component of educational and onboarding programs, with orientation and continuing education tailored to the distinct needs of different educational pathways rather than assuming a uniform baseline of documentation skills.

### 4.3. Workload

The nurses reported that excessive paperwork and electronic charting were mentally exhausting and time‐consuming. Although electronic documentation is intended to enhance efficiency and safety, participants described mixed experiences, reflecting a widely reported tension between the promise and reality of EHRs. Some acknowledged benefits, such as improved data quality, whereas others emphasized that poorly designed systems, technical disruptions, and insufficient training disrupted workflows. Technological tools, such as electronic records, aim to reduce errors and improve information flow; however, they may also increase workloads or alter workflows in unhelpful ways [38]. Prior evidence suggests that electronic documentation can save nursing time (by approximately 1.4 min per patient) and improve data quality [54]. However, technical difficulties, poor design, and limited training easily undermine these gains. Nursing staff depend on the usability and stability of technology to deliver care and ensure patient safety [55]. The broader literature has consistently linked documentation burden to nurse burnout, reduced bedside care time, and declining job satisfaction [56]. Research has shown that such burdens contribute to emotional exhaustion and impair the quality of patient care, as nurses struggle to balance competing priorities in fast‐paced environments [22].

### 4.4. Inconsistencies in Documentation Across Departments

Inconsistencies in documentation practices across units and departments further complicated the nurses’ experiences. Variations in charting formats, color coding, and electronic systems were perceived as barriers to effective communication, particularly during handovers and care transitions. Participants noted that this variability increased their cognitive load and heightened the risk of misinterpretation. Such inconsistency has been associated with inefficiencies, miscommunication, and patient safety incidents during transitions of care [57]. The absence of standardized documentation practices contributes to confusion and significantly impedes interdepartmental communication and continuity of care, consistent with evidence linking nonstandardized documentation to inefficiencies and safety risks [58]. Rather than reflecting individual shortcomings, these challenges point to structural and organizational factors that shape documentation environments.

### 4.5. Technical Limitations

Technical limitations, such as the physical placement of computers, frequent system downtimes, and inadequate access to electronic records at the point of care, further hindered timely and accurate documentation. These workflow barriers often compelled the nurses to rely on memory or make delayed entries, compromising data quality and clinical decision‐making. Using tablets has been shown to reduce the time spent on documentation [59], yet the absence of a user‐centered design in health information technology infrastructure may increase the cognitive load and reduce efficiency, particularly in high‐acuity settings [59]. Collectively, these findings highlight the need for harmonized, ergonomically designed electronic documentation systems that better support safe and efficient care delivery.

### 4.6. Leadership Responsiveness

A recurring concern was the perceived lack of responsiveness from leadership to frontline feedback. Several nurses, particularly those in junior roles, felt that their insights into documentation challenges were overlooked. This may contribute to reduced motivation, with documentation practices reported to be 59% lower among unmotivated nurses than among motivated nurses [5]. Such perceived disconnects can diminish engagement with documentation and weaken the perceived value of documentation standards. Recent research has emphasized that failing to incorporate frontline perspectives undermines staff morale and the effectiveness of quality improvement initiatives [60]. When nurses’ contributions are not acknowledged, it signals a lack of psychological safety and a weak participatory culture, both of which are essential for sustained improvements in documentation and patient care outcomes. These findings reflect broader organizational dynamics related to hierarchy, participation, and psychological safety. When nurses perceive limited opportunities to influence system design, documentation may be experienced as imposed rather than collaborative, potentially undermining its perceived legitimacy and usefulness.

Overall, the findings suggest that documentation practices are embedded within complex educational, technological, and organizational contexts. The study aligns with broader patient safety frameworks, including the WHO patient safety strategy. However, the findings should be interpreted as illustrative of local realities rather than definitive. The study contributes nuanced insights into how nurses experience documentation in practice and underscores the importance of aligning educational preparation, system design, and organizational culture to support safe and meaningful documentation. In the Saudi Arabian context, where substantial resources have been devoted to patient safety [47], evidence of ongoing gaps in proactive monitoring, learning, and system‐level integration [48] highlights the need to treat nursing documentation as a central component of patient safety infrastructure.

### 4.7. Implications and Recommendations

#### 4.7.1. Implications for Practice

This study identified several practice‐level priorities for nurse managers and clinical leaders. First, bridging training gaps requires integrating practical documentation competencies, particularly EHR use, into undergraduate programs, internships, and continuing professional development. Structured clinical EHR training can reduce documentation errors and improve nurses’ readiness for real‐world clinical settings [5]. Second, adopting standardized charting templates and harmonized EHR modules across departments can improve consistency and documentation quality, with structured formats shown to enhance data accuracy, completeness, and interpretability, thereby supporting safer clinical decision‐making [54]. Third, providing point‐of‐care documentation tools, such as mobile or bedside systems, can facilitate real‐time data entry and improve timeliness and accuracy. Successful implementation requires early nurse involvement, alignment with clinical workflows, and strong organizational support, including training and technical assistance [61]. Finally, reviewing and streamlining documentation requirements, together with optimizing EHR user interfaces, can help reduce documentation burden, mitigate burnout, and enable nurses to devote more time to direct patient care [56].

#### 4.7.2. Implications for Policy and Leadership

At the policy level, involving frontline nurses in the development and revision of documentation policies is essential to ensure that requirements are clinically realistic and perceived as meaningful. Participatory approaches foster a sense of ownership, enhance compliance, and improve user satisfaction, supporting sustainable practice change [54]. Policymakers and senior leaders should prioritize user‐centered documentation policies that balance regulatory and medico‐legal requirements with clinical practicality and workflow realities. Strengthening psychological safety and establishing formal mechanisms for feedback on documentation systems can signal that nurses’ experiential knowledge is valued, supporting motivation and engagement with documentation practices [5, 60]. For nursing management, these findings underscore the importance of visible, responsive leadership that treats documentation as a shared resource for safety and quality rather than a purely bureaucratic obligation.

#### 4.7.3. Implications for Future Research

Future research should examine the long‐term impact of standardized documentation systems and point‐of‐care technologies on patient safety outcomes, nurse workload, and burnout across diverse Saudi healthcare settings. Longitudinal and mixed‐method studies could clarify how documentation burden, organizational culture, and leadership responsiveness interact to shape care quality and staff retention. Evaluative studies exploring nurses’ experiences with policy‐driven documentation reforms would also provide valuable insights to inform national strategies for improving documentation quality, system usability, and the integration of documentation into broader patient safety and management initiatives.

### 4.8. Strengths and Limitations of the Study

To the best of our knowledge, this is the first qualitative study in Saudi Arabia that focuses on nursing documentation from the perspective of frontline nurses. One of the strengths of this study lies in its use of face‐to‐face interaction, which facilitated personal and comfortable discussions. However, the qualitative nature of this study and its small sample size limit the generalizability of the findings, as the results cannot be considered fully representative of the broader nursing population. Although data saturation was achieved, the relatively small sample size may limit the transferability of findings to all nursing contexts in Saudi Arabia. Future studies with larger, more diverse samples from various regions are needed to broaden the insights obtained in this study. To enhance the generalizability of the findings, quantitative methods should be employed in future research, along with a larger sample drawn from a broader range of healthcare institutions. Despite the limitations, efforts were made to ensure the credibility of the analysis, including collaborative discussions among the research teams to minimize bias in data interpretation. Interviews were conducted in Arabic and English, allowing participants to express themselves freely without language barriers. However, the process of transcribing, translating, and comparing audio recordings was intensive and posed methodological challenges.

## 5. Conclusion

This qualitative study explored Saudi nurses’ experiences with documentation tools and revealed three core themes and eight interconnected subthemes. The participants viewed documentation as essential for legal protection, communication, and patient safety; however, they reported frustration due to excessive paperwork, inadequate training, and technological inefficiencies. Most nurses initially perceived documentation as a legal safeguard but later recognized its value in ensuring patient safety. However, inconsistent systems, lack of standardization, and limited emphasis in the education and internship phases had left them underprepared. Although electronic systems improved data quality, their effectiveness was hindered by technical issues and poor design. Additionally, the nurses reported that their limited influence on policy development contributed to low motivation and reduced compliance with documentation requirements.

Our findings align with international research reporting that nurses often face inadequate training, inconsistent documentation practices, and burdensome systems that compromise the quality of care. Addressing these challenges requires systemic changes, including improved education, EHR optimization, staff inclusion, and workflow redesign, to enhance documentation quality, patient safety, and nurse well‐being. This study recommends closing existing gaps through curriculum reform, standardized templates, user‐friendly EHR tools, and active nurse involvement in the design of documentation policies. These strategies can improve accuracy, reduce burnout, and improve patient outcomes. Future research should adopt quantitative approaches and include more diverse samples to broaden the insights derived from this study.

## Funding

The authors disclose receipt of the following financial support for the research, authorship, and/or publication of this article: This study was funded by the Ongoing Research Funding Program (number: ORF‐2026‐1016) of King Saud University, Riyadh, Saudi Arabia. The funder had no role in the study design, data collection and analysis, decision to publish, or preparation of the manuscript.

## Conflicts of Interest

The authors declare no conflicts of interest.

## Data Availability

The data that support the findings of this study are available from the corresponding author upon reasonable request.
